# Altered filamin A enables amyloid beta-induced tau hyperphosphorylation and neuroinflammation in Alzheimer’s disease

**DOI:** 10.20517/2347-8659.2017.50

**Published:** 2017-12-08

**Authors:** Lindsay H. Burns, Hoau-Yan Wang

**Affiliations:** 1 Pain Therapeutics Inc., Austin, TX 78731, USA.; 2 Department of Physiology, Pharmacology and Neuroscience, City University of New York School of Medicine, New York, NY 10031, USA.; 3 Department of Biology and Neuroscience, Graduate School of the City University of New York, New York, NY 10031, USA.

**Keywords:** Proteopathy, hyperphosphorylation, α7 nicotinic acetylcholine receptor, toll-like receptor 4, neuroinflammation, PTI-125

## Abstract

Alzheimer’s disease (AD) is a neurodegenerative disease with proteopathy characterized by abnormalities in amyloid beta (Aβ) and tau proteins. Defective amyloid and tau propagate and aggregate, leading to eventual amyloid plaques and neurofibrillary tangles. New data show that a third proteopathy, an altered conformation of the scaffolding protein filamin A (FLNA), is critically linked to the amyloid and tau pathologies in AD. Altered FLNA is pervasive in AD brain and without apparent aggregation. In a striking interdependence, altered FLNA is both induced by Aβ and required for two prominent pathogenic signaling pathways of Aβ. Aβ monomers or small oligomers signal via the α7 nicotinic acetylcholine receptor (α7nAChR) to activate kinases that hyperphosphorylate tau to cause neurofibrillary lesions and formation of neurofibrillary tangles. Altered FLNA also enables a persistent activation of toll-like-receptor 4 (TLR4) by Aβ, leading to excessive inflammatory cytokine release and neuroinflammation. The novel AD therapeutic candidate PTI-125 binds and reverses the altered FLNA conformation to prevent Aβ’s signaling via α7nAChR and aberrant activation of TLR4, thus reducing multiple AD-related neuropathologies. As a regulator of Aβ’s signaling via α7nAChR and TLR4, altered FLNA represents a novel AD therapeutic target.

## INTRODUCTION

Alzheimer’s disease (AD) is a complex neurodegenerative disease characterized by a variety of synaptic and receptor dysfunctions, neuroinflammation, insulin resistance, degeneration and atrophy. Although the pathogenesis of AD is debated, the disease itself can be considered a proteopathy, a disease of abnormal proteins, due to the misfolding and aggregation of amyloid beta (Aβ) and hyperphosphorylated tau in brain areas critical to cognition and memory. These abnormal proteins ultimately form the histopathological hallmarks of AD brain: amyloid plaques and tau-containing tangles. Misfolded dysfunctional proteins and their aggregation occurs in many other neurodegenerative diseases including Parkinson’s disease, dementia with Lewy bodies, multiple systems atrophy, frontotemporal dementia, amyotrophic lateral sclerosis and Huntington’s disease^[1–4]^. Typically, misfolded proteins self-aggregate, creating intracellular inclusions that become extracellular deposits following cell death. In many cases, misfolded proteins propagate in a cell-to-cell “prion-like” manner^[1–3,5]^.

The proteopathy of Aβ in AD is an amyloidosis, meaning the protein converts from an α-helix-rich state to a β-sheet conformation. To enter an amyloid or β-sheet state, proteins must expose the backbone amide N-H and C=O groups to allow hydrogen bonding^[4]^. Cleavage of amyloid precursor protein into Aβ_42_ by secretases can expose these amide N-H and C=O groups and promote a β-sheet conformation^[4]^. Elevated concentrations from overproduction or insufficient clearance/processing may also contribute. The hydrogen bonding of the pleated, β-sheet conformation between parallel or anti-parallel β-sheets is much stronger than that in native α-helices, making reversal unlikely. Additionally, the β-sheet conformation allows hydrogen bonding between separate, stacked molecules, promoting oligomerization and eventual plaque formation^[4]^. Aβ is proposed to form a toxic, small oligomer “seed” requiring 3 or 4 molecules used as a template to “infect” native molecules and propagate in a prion-like manner^[6]^.

The proteopathy of tau, or tauopathy, in AD is primarily caused by hyperphosphorylation. Hyperphosphorylated tau loses its function of stabilizing microtubules and dissociates from them^[7]^. The increased pool of free tau after dissociation from microtubules is likely an important first step to aggregation in AD^[8]^. Untethered from microtubules, hyperphosphorylated tau twists together to form the paired helical filaments (PHFs) found in neurofibrillary tangles. In a toxic gain of function, hyperphosphorylated tau also actively disrupts microtubules and inhibits their assembly^[7,9]^ and even sequesters functional tau and other microtubule associated proteins^[9]^. Hyperphosphorylation also changes tau’s localization from axon-predominant to include dendrites, neuronal cell bodies and presynaptic areas, leading to synaptic dsyfunction^[10–12]^.

This mini-review focuses on a third, interconnected proteopathy in AD and its critical role in 2 toxic signaling pathways of soluble Aβ. The newly described proteopathy is an altered conformation of the ubiquitous scaffolding protein filamin A (FLNA), induced by Aβ_42_ and without apparent aggregation^[13]^. The 1st toxic cascade of Aβ enabled by altered FLNA is Aβ’s signaling via α7 nicotinic acetylcholine receptor (α7nAChR) to hyperphosphorylate tau. The 2nd signaling pathway is Aβ’s aberrant activation of toll-like-receptor 4 (TLR4), by binding CD14^[14]^, to induce neuroinflammation. In both cases, altered FLNA associates with these receptors to allow their aberrant signaling by Aβ^[13,15]^. Native FLNA in control brains does not associate with either receptor. Whether the altered conformation precedes or is a consequence of these receptor linkages is discussed. The interdependence of altered FLNA with Aβ signaling to hyperphosphorylate tau and promote neuroinflammation has been elucidated via the reversal of the FLNA proteopathy by a small molecule therapeutic candidate, PTI-125.

## Aβ SIGNALING VIA α7NACHR TO HYPERPHOSPHORYLATE TAU

The most toxic form of Aβ is considered soluble Aβ oligomers rather than plaque deposits^[16,17]^. Evidence that soluble Aβ induces tau pathology has grown, with hyperphosphorylation as the primary pathological modification^[18]^. Extensive research has elucidated the role of the α7nAChR in the toxicity of soluble Aβ_42_ and the consequent hyperphosphorylation of tau^[19–24]^. Soluble Aβ_42_ in monomeric or oligomeric form binds and signals via α7nAChR^[25–28]^, essentially hijacking this receptor to abnormally activate various kinases^[27,29–31]^ to heighten tau phosphorylation. Supportive data include co-localization of Aβ_42_ and α7nAChR in AD pyramidal neurons and a complete blockade of Aβ_42_-induced tau hyperphosphorylation *in vitro* by α7nAChR antisense oligonucleotides^[32]^. Aβ_42_ dose-dependently activates tau kinases to persistently phosphorylate tau at the three proline-directed sites, resulting in elevated hyperphosphorylated tau in neurofibrillary tangles. This Aβ-driven tau hyperphosphorylation can also be blocked by the α7nAChR antagonist α-bungarotoxin or other α7nAChR ligands if administered prophylactically^[32–35]^. The hyperphosphorylation of tau renders it dysfunctional, alters its cellular distribution and disrupts axonal/dendritic transport, leading to neurofibrillary lesions, dendritic breakdown, and ultimately, neurofibrillary tangles^[27]^. Importantly, soluble Aβ_42_ binds α7nAChR with an extraordinarily high (high femtomolar) affinity, rendering the Aβ_42_-α7nAChR interaction nearly irreversible^[26,36]^.

Though other targets have been demonstrated for soluble Aβ_42_, their nanomolar or lower affinities suggest high off-rates and limited target engagement, in contrast to Aβ_42_’s nearly irreversible sub-picomolar interaction with α7nAChR. Other targets of soluble Aβ include PrP^C^, a prion receptor, which Aβ binds at 50–100 nmol/L to suppress LTP in slice cultures^[37]^. Acting as a co-receptor for the Aβ-PrP^c^ complex, mGluR5 also plays a role in the impaired LTP^[38]^. Soluble Aβ has also been shown to bind neuroligin-1, a postsynaptic cell adhesion protein, in the nanomolar range, and this interaction has been proposed to promote Aβ oligomer formation^[39]^.

Evidence that phosphorylation can alter tau’s conformation comes from a study showing that a particular AD-specific phosphorylated tau species is only formed when specific phosphoepitopes in a proline-rich region are sequentially phosphorylated by GSK-3β (at Thr212) and then by PKA (at Ser214)^[40]^. Whether tau’s hyperphosporylation contributes to protein misfolding prior to formation of PHFs is speculative, particularly as its pathological structure has not been elucidated. The tau proteopathy in AD, therefore, involves hyperphosphorylation, but may or may not include misfolding. The formation of PHFs requires hyperphosphorylated tau, and tau protein in neurofibrillary tangles is hyperphosphorylated, most notably at Ser^202^, Thr^231^ and Thr^181[41]^. Interestingly, the alpha-synuclein that forms fibrils and is abundant in Lewy bodies in Parkinson’s disease is also hyperphosphorylated, at a single serine site^[42]^.

## ALTERED FLNA LINKS Aβ AND TAU PROTEOPATHIES

**Figure 1:**
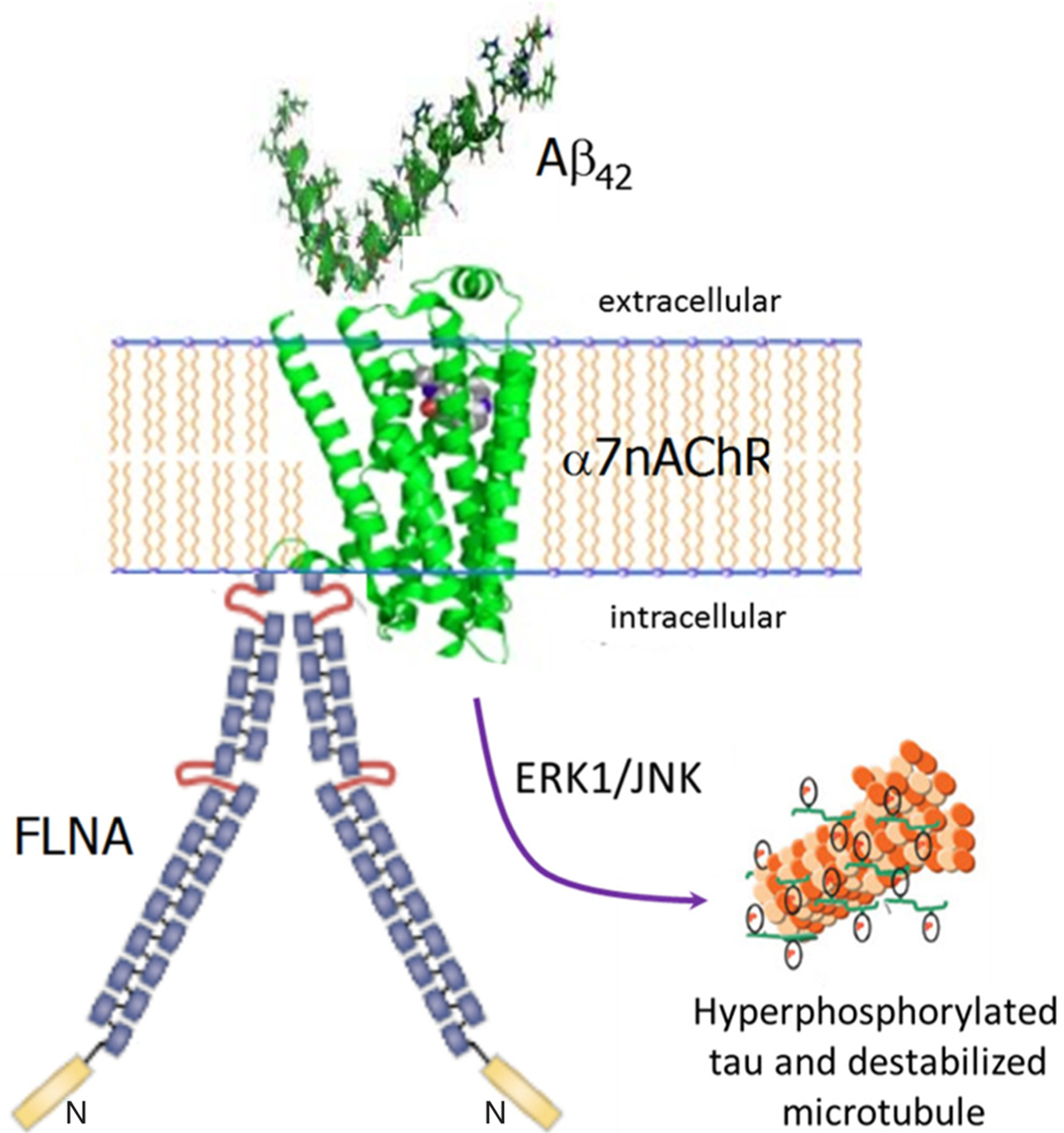
Altered FLNA linkage to α7nAChR enables Aβ_42_’s toxic signaling via α7nAChR to hyperphosphorylate tau. Monomers or small oligomers of Aβ_42_ bind α7nAChR, which recruits FLNA to link to α7nAChR. This recruitment likely alters FLNA’s conformation, which in turn increases the affinity of the Aβ_42_-α7nAChR interaction to a femtomolar affinity and enables the signaling. ERK1 and JNK kinases are activated to hyperphosphorylate tau. Hyperphosphorylated tau loses its function of stabilizing microtubules and dissociates from them, eventually creating PHFs and neurofibrillary tangles. FLNA: filamin A; Aβ: amyloid beta; α7nAChR: α7 nicotinic acetylcholine receptor; PHF: paired helical filament

We recently described a third, atypical proteopathy in AD that is critically interconnected with the toxicities of both Aβ_42_ and tau. This third proteopathy is an altered conformation of the scaffolding protein FLNA. It is induced by Aβ_42_, and in reciprocal action, enables Aβ_42_’s toxic signaling via α7nAChR to activate kinases that hyperphosphorylate tau^[13]^. Altered FLNA enables Aβ_42_’s signaling via α7nAChR by associating with this receptor^[13,15]^. Although FLNA constitutively associates with other receptors including the mu opioid receptor and insulin receptors^[43]^, FLNA does not normally link to α7nAChR. We hypothesize that upon Aβ_42_ binding to α7nAChR, FLNA is recruited to this receptor and its conformation altered to enable Aβ_42_’s aberrant signaling [Figure 1]. FLNA in control brains can be induced to link to α7nAChR by incubation with Aβ_42_ *in vitro* or by ICV Aβ_42_ infusion *in vivo*^[13,15]^. The altered conformation of FLNA is also illustrated by the 100-fold difference in binding affinities of the novel drug candidate PTI-125 to FLNA immunopurified from human postmortem AD *vs*. control brain^[13]^. One distinct difference between altered FLNA and other proteopathies is that the altered conformation of FLNA does not appear to promote self-aggregation.

Best known for cross-linking actin to enable cell structure, flexibility and motility, FLNA is a prominent regulator of the actin cytoskeletal assembly and dynamics^[44–46]^. The actin cytoskeleton, a vital component in synapses and the dendritic network, is impaired in AD^[47]^. Hence, the FLNA proteopathy might also disrupt synaptic and dendritic function in AD by disrupting actin dynamics. FLNA exists as an intracellular homodimer and dimerizesin a membrane-bound, C-terminal domain. It is a large (280-kDa) protein with 24 immunoglobulin repeats that are natively β-sheet pleated, forming two rod-like domains separated by two hinge regions. The nature of the conformational change of FLNA in AD is not yet known, thoughit is interesting that native FLNA is predominantly β-sheeted. Demonstrating induction by Aβ, the altered FLNA conformation exists not only in postmortem human AD brain and in triple transgenic (3xTg) AD mice, but also in ICV Aβ_42_-infused wildtype mice and in Aβ_42_-treated postmortem human control brain^[13]^.

This altered form of FLNA was evidenced by a shift in isoelectric focusing point (pI) from 5.9 in the native state to 5.3 in postmortem human AD brain or brains of mouse models^[13]^. An altered pI can indicate an altered conformation, reflecting changes in hydrogen bonding, charge-charge interactions or accessibility of ionizable residues within the molecule^[48–50]^. In this case, the shifted pI is resistant to complete dephosphorylation by alkaline phosphatase. Hence, unlike the proteopathies of tau and alpha-synuclein, the altered conformation of FLNA is not due to changes in phosphorylation state. Further studies are needed to reveal the details of FLNA’s conformational change and whether altered FLNA is unique to AD.

The induction of the altered conformation by Aβ_42_ may suggest a direct interaction or some sort of cross-protein templating by Aβ_42_. However, Aβ_42_ and FLNA do not directly interact because neither protein can be co-immunoprecipitated with the other (our unpublished observations). Although it is tempting to presume that FLNA must be in its altered conformation to associate with α7nAChR, we hypothesize that it is FLNA’s transmembrane recruitment to this receptor, induced by Aβ_42_’s extracellular binding, that changes FLNA’s conformation. Aβ_42_ binds α7nAChR with high femtomolar affinity, the highest known binding affinity of Aβ_42_. Interestingly, prevention of the FLNA linkage to α7nAChR by FLNA-binding compound PTI-125 decreases Aβ_42_’s affinity for this receptor 1,000–10,000-fold, illustrating that FLNA enables not only Aβ_42_’s toxic signaling but also its high-affinity binding for α7nAChR^[15]^. Although this observation appears to suggest that altered FLNA is responsible for Aβ_42_’s binding, we propose a dynamic, sequential process: (1) Aβ_42_ binds α7nAChR to induce FLNA recruitment; (2) recruitment alters FLNA’s conformation; and (3) FLNA’s altered form secures (locks in) an ultra-high affinity Aβ_42_-α7nAChR interaction. The reasoning behind this hypothesis is that Aβ_42_ induces both the aberrant FLNA conformation and its recruitment to α7nAChR (either by ICV Aβ_42_ infusion to wildtype mice or by *in vitro* Aβ_42_ incubation of postmortem control brain)^[13,15]^ and that Aβ_42_ and FLNA do not themselves interact.

Enabled by FLNA, Aβ_42_, in monomeric or small oligomeric form^[51]^, signals via α7nAChR to activate kinases to hyperphosphorylate tau protein^[30,32,33,52]^. We know that the linkage of altered FLNA is required for this Aβ signaling pathway because the novel drug candidate PTI-125 prevents the FLNA-α7nAChR linkage and greatly reduces tau hyperphosphorylation^[13,15]^. Hyperphosphorylated tau loses its ability to stabilize microtubules and dissociates from them, thereby increasing the pool of free phosphorylated tau that eventually appears in neurofibrillary tangles. We suggest that the signaling of Aβ_42_ via α7nAChR contributes prominently to a variety of AD-related neuropathologies in addition to, or perhaps elicited by, tau hyperphosphorylation because these additional neuropathologies are also reduced by PTI-125’s disruption of Aβ_42_’s signaling via α7nAChR^[13,15]^. Alternatively, they may be related to the neuroinflammatory signaling that is also disrupted by PTI-125 as discussed below. An obvious consequence of the toxic signaling through α7nAChR is that the normal function of α7nAChR is impaired. Because α7nAChR is an upstream regulator of the N-methyl-D-aspartate receptor (NMDAR)^[53,54]^, the impaired NMDAR signaling in AD brain and 3xTg AD mice is very likely related to the impaired signaling via α7nAChR. This assertion is supported by the improvement in signaling function of both receptors by PTI-125^[13,15]^. Aβ_42_’s signaling to hyperphosphorylate tau also contributes to eventual formation of Aβ deposits and tau-containing neurofibrillary lesions^[55,56]^, because disrupting this signaling via PTI-125 markedly reduces both Aβ deposits and neurofibrillary lesions in 3xTg AD mice or ICV Aβ_42_-infused wildtype mice^[13,15]^. Finally, reiterating the improved receptor function by PTI-125 and implicating this toxic Aβ signaling pathway in cognitive impairment, PTI-125 also improved working and spatial memory and nesting behavior abnormalities in 3xTg AD mice^[13]^.

## ALTERED FLNA ENABLES Aβ-INDUCED NEUROINFLAMMATION

**Figure 2:**
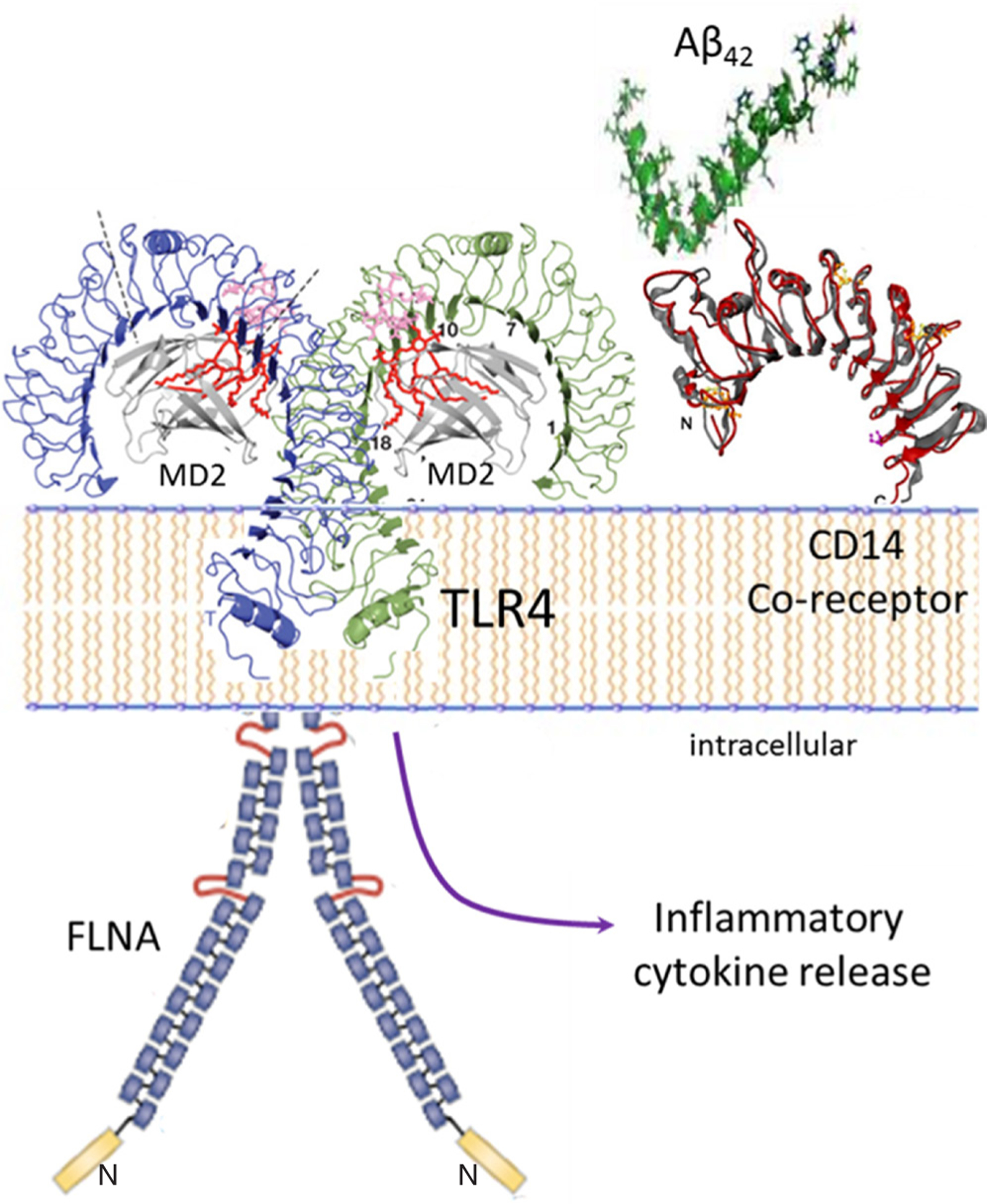
Altered FLNA linkage to TLR4 enables persistent Aβ_42_-induced TLR4 activation and neuroinflammation. Aβ_42_ binds the CD14 co-receptor to induce FLNA recruitment to TLR4. As with α7nAChR, the FLNA linkage likely alters the FLNA conformation. With Aβ_42_ binding CD14, altered FLNA linkage to TLR4 enables a sustained TLR4 activation, leading to substantial inflammatory cytokine release and the neuroinflammation characteristic of AD. FLNA: filamin A; Aβ: amyloid beta; α7nAChR: α7 nicotinic acetylcholine receptor; TLR4: toll-like-receptor 4

The proteopathy of FLNA enables a second toxic signaling pathway of Aβ: its activation of the innate immune receptor TLR4^[13,15]^. Aβ_42_ binds the CD14 co-receptor^[14]^, complexed with TLR4, to induce an aberrant FLNA linkage to TLR4^[13,15]^, which appears to reciprocally enable a sustained Aβ-mediated TLR4 activation [Figure 2]. Hence, the FLNA-TLR4 linkage, allowing Aβ activation of TLR4, promotes persistent inflammatory cytokine release and elicits neuroinflammation characteristic of AD. As with α7nAChR, native FLNA in control brains does not associate with TLR4, unless treated with Aβ_42_ by ICV infusion to wildtype mice or by *in vitro* incubation of control postmortem human brain^[13,15]^. Like the FLNA recruited to α7nAChR, FLNA that links to TLR4 in the presence of Aβ_42_, or in AD postmortem brain or 3xTg AD mice, has an altered conformation^[13]^. The question arises again whether Aβ_42_ binding to CD14 induces the FLNA linkage to TLR4 and the altered FLNA conformation in the process, or whether this linkage happens only after FLNA’s conformation is altered. With two receptors seemingly controlling FLNA’s conformation by recruitment, it is tempting to speculate that one linkage or the other induces the altered conformation, unleashing further aberrant and persistent receptor associations with FLNA. Notably, even in its non-diseased state, FLNA interacts with more than 90 different protein partners including a few receptors and many signaling molecules^[44]^. With multiple interactions, it is possible that one or more specific protein interactions - induced by Aβ - could alter FLNA’s conformation and subsequent behavior. If so, it is then possible that the alteration in FLNA could also affects its interaction with other protein partners and their functions, disrupting the integrity of circuitries to propagate dysfunction in AD brains.

## REVERSING FLNA’S ALTERED CONFORMATION

**Figure 3:**
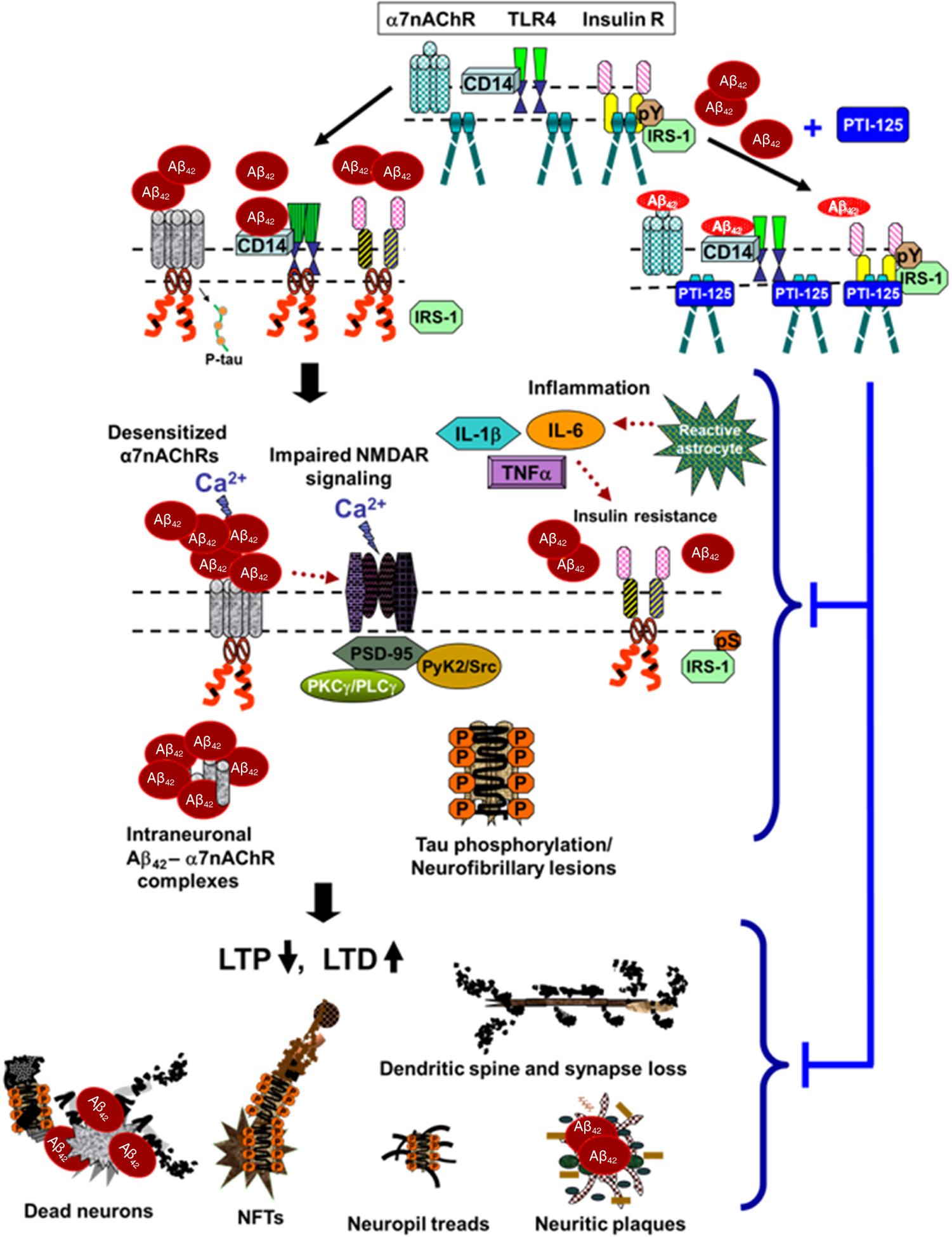
Proposed model of pathological consequences of altered FLNA-enabled Aβ_42_ signaling via α7nAChR and TLR4. Soluble Aβ_42_ monomers or small oligomers bind α7nAChR or CD14, complexed with TLR4, inducing recruitment of FLNA to these receptors. Dimers of native FLNA, coupled to insulin receptors but not to α7nAChR or TLR4, are depicted as straight rods; red curly FLNA depicts the altered form, which is recruited to α7nAChR and TLR4 (and possibly also insulin receptors). Enabled by altered FLNA’s new linkages, Aβ_42_ activates α7nAChR to hyperphosphorylate tau and persistently activates TLR4 to induce inflammatory cytokine release (TNFα, IL-1β and IL-6) by reactive astrocytes. This neuroinflammation likely contributes to insulin receptor desensitization^[57]^. Although the insulin receptor is constitutively associated with native FLNA, it is possible that altered FLNA also contributes to the insulin receptor dysfunction in AD. Aβ_42_’s aberrant signaling through α7nAChR impairs function of α7nAChR and of NMDARs, restricting calcium influx through both receptors. Increasing Aβ_42_ piling onto α7nAChR leads to intraneuronal Aβ_42_-α7nAChR complexes. The hyperphosphorylated tau dissociates from microtubules, disrupting microtubule stability, axonal transport and neuronal function. Along with dysfunctional tau, impaired NMDARs reduce LTP and heighten LTD. Dendritic spines and synapses are lost. Neuritic plaques, neuropil treads and neurofibrillary tangles are formed, and neurons degenerate. FLNA: filamin A; Aβ: amyloid beta; α7nAChR: α7 nicotinic acetylcholine receptor; TLR4: toll-like-receptor 4; TNFα: tumor necrosis factor-α; IL: interleukin; AD: Alzheimer’s disease; NMDAR: N-methyl-D-aspartate receptor; LTP: long-term potentiation; LTD: long-term depression

The primary evidence for the role of FLNA’s altered conformation in Aβ_42_’s toxic signaling via α7nAChR and TLR4 comes from the small molecule therapeutic candidate PTI-125. The altered conformation of FLNA was first inferred from the efficacy (as well as safety) at relatively low doses of PTI-125 in mouse models and *in vitro*, despite a ubiquitous target. We subsequently determined that PTI-125 binds altered FLNA (in AD postmortem brain, in 3xTg AD mice or in ICV Aβ_42_-infused mice) much more tightly than native FLNA (in control postmortem brain or in control mice): a femtomolar affinity was measured for altered FLNA *vs*. a substantially lower, picomolar affinity for native FLNA^[13]^. Using the same isoelectric focusing point assessment that demonstrated FLNA’s altered conformation, we further showed that PTI-125 binding, by *in vivo* treatment of mice or by *in vitro* incubation of postmortem AD brain, restores the native conformation of FLNA^[13]^. By reversing the FLNA proteopathy, PTI-125 dramatically reduces FLNA’s aberrant linkages to both α7nAChR and TLR4, consequently reducing tau hyperphosphorylation and neuroinflammation^[13,15]^. By attenuating Aβ’s pathogenic drive, PTI-125 treatment improved function of three key receptors: α7nAChR, NMDAR and insulin receptors^[13,15]^. The improved insulin receptor signaling may also reflect reduced neuroinflammation by PTI-125, as neuroinflammation has been linked to impaired insulin receptor function^[57]^. Disrupting the FLNA-TLR4 linkage, PTI-125 potently and efficaciously reduced inflammatory cytokines by at least 80% in 3xTg AD mice and in ICV Aβ_42_-infused mice^[13,15]^. Correlating with the improved NMDAR function, synaptic plasticity was also improved by PTI-125 treatment of either 3xTg AD mice or Aβ_42_-treated postmortem human control brain, evidenced by improved activity-dependent expression of the master synaptic plasticity regulator, activity-regulated, cytoskeleton-associated protein (Arc)^[13]^. These beneficial effects of PTI-125 in AD mouse models and postmortem AD brain elucidate a critical role of the FLNA proteopathy in multiple toxicities of Aβ and tau, including neuroinflammation [Figure 3]. We also speculate that altered FLNA may alter actin dynamics, given the primary role of FLNA in the actin cytoskeleton.

## LIMITATIONS OF INTERPRETATION

There are limitations to our interpretations of our data. First, the alteration in FLNA has not been directly demonstrated. It is implied by a shift in pI for FLNA in AD *vs*. control brain and supported by the differential binding affinities of PTI-125 to FLNA in control *vs*. AD brain. Representing an aggregate pKa for all amino acids in a protein, a shifted pI is assumed to reflect a change in structure. Second, the possibility exists that PTI-125 has additional significant targets besides FLNA’s altered conformation that have confounded our interpretation of its effects. We believe this is a remote possibility because: (1) PTI-125 showed no interactions in a lead profiling screen of 68 receptors, channels and transporters; (2) the binding affinity of PTI-125 in AD brain was virtually identical to its binding affinity for FLNA immunopurified from AD brain^[13]^, indicating a lack of other CNS targets; and (3) PTI-125 shows good CNS penetration and more than sufficient brain levels for a femtomolar target.

Both Aβ and tau as AD therapeutic targets have been questioned following multiple clinical trial failures, and this skepticism may extend to altered FLNA as a viable therapeutic target for AD. Furthermore, several partial agonists or positive allosteric modulators to α7nAChR have also failed, challenging the significance of this Aβ signaling pathway. As commonly suggested, potential reasons for these failures include treating too late in disease progression, because neuropathology precedes symptoms by 10–25 years^[58,59]^, and the possible protective effect of amyloid^[60]^. Additionally, agents that target aggregation of Aβ or tau may be downstream of significant dysfunction, as suggested by the pathways discussed here. Perhaps most importantly, the femtomolar interaction of Aβ_42_ with α7nAChR imposes substantial competition for antibodies or small molecules seeking to compete directly with this interaction.

An additional caution to interpretation of our data may be our use of synthetic, monomeric Aβ_42_ rather than oligomeric Aβ, thought to be the most toxic Aβ species. Though transgenic mice generate both monomeric and oligomeric Aβ, Aβ_42_ *in vitro* forms a mixture of monomers and small oligomers as well, and both signal via α7nAChR^[51]^.

Finally, despite the broad spectrum of beneficial effects demonstrated preclinically by PTI-125’s reversal of FLNA’s altered conformation, Aβ_42_ signaling via α7nAChR and TLR4 are only two of many pathogenic cascades in AD. A variety of other approaches have demonstrated similar results in mouse models, and the difficulty of clinical translation remains. We believe our strongest data validating FLNA’s altered conformation as a novel target for AD drug development are those using postmortem human AD or Aβ_42_-treated control brainwith postmortem interval < 10 h. PTI-125 demonstrated efficacy in postmortem human brain at concentrations as low as 1 pmol/L^[13]^.

## CONCLUSION

Abnormal FLNA is intertwined with the Aβ and tau proteopathies in AD. Aβ_42_ induces the altered conformation of FLNA, and altered FLNA enables sustained Aβ_42_ signaling via α7nAChR and TLR4, possibly by securing high affinity binding of Aβ^[13,15]^. Aβ_42_’s toxic signaling via α7nAChR activates kinases to hyperphosphorylate tau^[25–31]^, leading to its dysfunction and aggregation, and the FLNA-enabled Aβ42 activation of TLR4 enhances neuroinflammation^[13,15]^. Moreover, the altered conformation of FLNA is an important catalyst of Aβ toxicities and tau dysfunction, including neuroinflammation and synaptic dysfunction. The novel therapeutic candidate PTI-125 offers the possibility of dampening these toxic cascades by restoring the native conformation of FLNA. In addition to its novel target, preferentially binding and reversing an altered protein conformation is a novel mechanism of action for any drug candidate. An advantage of its mechanism is that PTI-125 dramatically attenuates Aβ’s aberrant activation of both α7nAChR and TLR4 without directly affecting these surface receptors. PTI-125 preserves tau’s neuronal function, preserving axonal transport, and reduces Aβ-induced neuroinflammation and synaptic/dendritic damage while maintaining health of the receptors. AD is a disease of multiple dysfunctions. Interconnected with several toxicities of Aβ and tau and implicated in many AD-related neuropathologies, the proteopathy of FLNA represents an entirely new target for AD drug development.

## References

[1] Frost B, Diamond M. Prion-like mechanisms in neurodegenerative diseases. Nat Rev Neurosci 2010;11:155–9. 20029438 10.1038/nrn2786 PMC3648341

[2] Sengupta U, Nilson A, Kayed R. The role of amyloid-β oligomers in toxicity, propagation, and immunotherapy. EBioMedicine 2016;6:42–9. 27211547 10.1016/j.ebiom.2016.03.035 PMC4856795

[3] Yin R, Tan L, Jiang T, Yu J. Prion-like mechanisms in Alzheimer’s disease. Curr Alzheimer Res 2014;11:755–64. 25212914 10.2174/156720501108140910121425

[4] Eisenberg D, Jucker M. The amyloid state of proteins in human diseases. Cell 2012;148:1188–203. 22424229 10.1016/j.cell.2012.02.022 PMC3353745

[5] Rasmussen J, Jucker M, Walkerd L. Aβ seeds and prions: how close the fit? Prion 2017;11:215–25. 28657440 10.1080/19336896.2017.1334029 PMC5553305

[6] Nelson R, Sawaya M, Balbirnie M, Madsen A, Riekel C, Grothe R, Eisenberg D. Structure of the cross-beta spine of amyloid-like fibrils. Nature 2005;435:773–8. 15944695 10.1038/nature03680 PMC1479801

[7] Alonso A, Zaidi T, Grundke-Iqbal I, Iqbal K. Role of abnormally phosphorylated tau in the breakdown of microtubules in Alzheimer disease. PNAS 1994;91:5562–6. 8202528 10.1073/pnas.91.12.5562 PMC44036

[8] Friedhoff P, von Bergena M, Mandelkowa EM, Mandelkow E. Structure of tau protein and assembly into paired helical filaments. Biochimica et Biophysica Acta 2000;1502:122–32. 10899437 10.1016/s0925-4439(00)00038-7

[9] Alonso A, Grundke-Iqbal I, Barra H, Iqbal K. Abnormal phosphorylation of tau and the mechanism of Alzheimer neurofibrillary degeneration: sequestration of microtubule-associated proteins 1 and 2 and the disassembly of microtubules by the abnormal tau. PNAS 1997;94:298–303. 8990203 10.1073/pnas.94.1.298 PMC19321

[10] Hoover B, Reed M, Su J, Penrod R, Kotilinek L, Grant M, Pitstick R, Carlson G, Lanier L, Yuan L, Ashe K, Liao D. Tau mislocalization to dendritic spines mediates synaptic dysfunction independently of neurodegeneration. Neuron 2010;68:1067–81. 21172610 10.1016/j.neuron.2010.11.030 PMC3026458

[11] Ittner L, Ke Y, Delerue F, Bi M, Gladbach A, van Eersel J, Wölfing H, Chieng B, Christie M, Napier I, Eckert A, Staufenbiel M, Hardeman E, Götz J. Dendritic function of tau mediates amyloid-beta toxicity in Alzheimer’s disease mouse models. Cell 2010;142:387–97. 20655099 10.1016/j.cell.2010.06.036

[12] Kowall N, Kosik K. Axonal disruption and aberrant localization of tau protein characterize the neuropil pathology of Alzheimer’s disease. Ann Neurol 1987;22:639–43. 3122646 10.1002/ana.410220514

[13] Wang HY, Lee KC, Pei Z, Khan A, Bakshi K, Burns L. PTI-125 binds and reverses an altered conformation of filamin A to reduce Alzheimer’s disease pathogenesis. Neurobiol Aging 2017;55:99–114. 28438486 10.1016/j.neurobiolaging.2017.03.016

[14] Gambuzza M, Sofo V, Salmeri F, Soraci L, Marino S, Bramanti P. Toll-like receptors in Alzheimer’s disease: a therapeutic perspective. CNS Neurol Disord Drug Targets 2014;13:1542–58. 25106635 10.2174/1871527313666140806124850

[15] Wang H-Y, Bakshi K, Frankfurt M, Stucky A, Goberdhan M, Shah S, Burns L. Reducing amyloid-related Alzheimer’s disease pathogenesis by a small molecule targeting filamin A. J Neurosci 2012;32:9773–84. 22815492 10.1523/JNEUROSCI.0354-12.2012 PMC6621293

[16] Näslund J, Haroutunian V, Mohs R, Davis K, Davies P, Greengard P, Buxbaum J. Corrleation between elevated levels of amyolid beta-peptide in the brain and cognitive decline. JAMA 2000;283:1571–7. 10735393 10.1001/jama.283.12.1571

[17] Gandy S, Simon A, Steele J, Lublin A, Lah J, Walker L, Levey A, Krafft G, Levy E, Checler F, Glabe C, Bilker W, Abel T, Schmeidler J, Ehrlich M. Days-to-criterion as an indicator of toxicity associated with human Alzheimer amyloid-β oligomers. Ann Neurol 2010;68:220–30. 20641005 10.1002/ana.22052 PMC3094694

[18] Stancu I-C, Vasconcelos B, Terwel D, Dewachter I. Models of β-amyloid induced Tau-pathology: the long and “folded” road to understand the mechanism. Mol Neurodegen 2014;9:51. 10.1186/1750-1326-9-51 PMC4255655 25407337

[19] Medeiros R, Castello N, Cheng D, Kitazawa M, Baglietto-Vargas D, Green K, Esbenshade T, Bitner R, Decker M, LaFerla F. α7 Nicotinic receptor agonist enhances cognition in aged 3xTg-AD mice with robust plaques and tangles. Am J Pathol 2014;184:520–9. 24269557 10.1016/j.ajpath.2013.10.010

[20] Inestrosa N, Godoy J, Vargas J, Arrazola M, Rios J, Carvajal F, Serrano F, Farias G. Nicotine prevents synaptic impairment induced by amyloid-β oligomers through α7-nicotinic acetylcholine receptor activation. Neuromolecular Med 2013;15:549–69. 23842742 10.1007/s12017-013-8242-1

[21] Dziewczapolski G, Glogowski C, Masliah E, Heinemann S. Deletion of the alpha 7 nicotinic acetylcholine receptor gene improves cognitive deficits and synaptic pathology in a mouse model of Alzheimer’s disease. J Neurosci 2009;29:8805–15. 19587288 10.1523/JNEUROSCI.6159-08.2009 PMC2753494

[22] Ni R, Marutle A, Nordberg A. Modulation of α7 nicotinic acetylcholine receptor and fibrillar amyloid-β interactions in Alzheimer’s disease brain. J Alzheimers Dis 2013;33:841–51. 23042213 10.3233/JAD-2012-121447

[23] Ondrejcak T, Wang Q, Kew J, Virley D, Upton N, Anwyl R, Rowan M. Activation of α7 nicotinic acetylcholine receptors persistently enhances hippocampal synaptic transmission and prevents Aβ-mediated inhibition of LTP in the rat hippocampus. Eur J Pharmacol 2012;677:63–70. 22200627 10.1016/j.ejphar.2011.12.008

[24] D’Andrea M, Nagele R. Targeting the alpha 7 nicotinic acetylcholine receptor to reduce amyloid accumulation in Alzheimer’s disease pyramidal neurons. Curr Pharm Des 2006;12:677–84. 16472157 10.2174/138161206775474224

[25] Tong M, Arora K, White M, Nichols R. Role of key aromatic residues in the ligand-binding domain of alpha7 nicotinic receptors in the agonist action of beta-amyloid. J Biol Chem 2011;286:34373–81. 21828053 10.1074/jbc.M111.241299 PMC3190827

[26] Wang H, Lee D, Davis C, Shank R. Amyloid peptide Abeta(1–42) binds selectively and with picomolar affinity to alpha7 nicotinic acetylcholine receptors. J Neurochem 2000;75:1155–61. 10936198 10.1046/j.1471-4159.2000.0751155.x

[27] Wang H, Li W, Benedetti N, Lee D. α7 Nicotinic acetylcholine receptors mediate β-amyloid peptide-induced tau protein phosphorylation. J Biol Chem 2003;278:31547–53. 12801934 10.1074/jbc.M212532200

[28] Wang HY LD, D’Andrea MR, Peterson PA, Shank RP, Reitz AB. beta-Amyloid(1–42) binds to alpha7 nicotinic acetylcholine receptor with high affinity. Implications for Alzheimer’s disease pathology. J Biol Chem 2000;275:5626–32. 10681545 10.1074/jbc.275.8.5626

[29] Dineley K, Bell K, Bui D, Sweatt J. β-Amyloid peptide activates α7 nicotinic acetylcholine receptors expressed in xenopus oocytes. J Biol Chem 2002;277:25056–61. 11983690 10.1074/jbc.M200066200

[30] Hu M, Waring J, Gopalakrishnan M, Li J. Role of GSK-3beta activation and alpha7 nAChRs in Abeta(1–42)-induced tau phosphorylation in PC12 cells. J Neurochem 2008;106:1371–7. 18485099 10.1111/j.1471-4159.2008.05483.x

[31] Zhang L, Xie J, Yang J, Cao Y. Tyrosine phosphatase STEP61 negatively regulates amyloid β-mediated ERK/CREB signaling pathways via α7 nicotinic acetylcholine receptors. J Neurosci Res 2013;91:1581–90. 24123152 10.1002/jnr.23263

[32] Wang HY, Li W, Benedetti N, Lee D. α7 nicotinic acetylcholine receptors mediate β-amyloid peptide-induced tau protein phosphorylation. J Biol Chem 2003;278:31547–53. 12801934 10.1074/jbc.M212532200

[33] Dineley K, Bell K, Bui D, Sweatt J. β-Amyloid peptide activates α7 nicotinic acetylcholine receptors expressed in xenopus oocytes. J Biol Chem 2002;227:25056–61. 10.1074/jbc.M200066200 11983690

[34] Wang HY, Bakshi K, Shen C, Frankfurt M, Trocme-Thibierge C, Morain P. S 24795 limits β-amyloid - α7 nicotinic receptor interaction and reduces Alzheimer’s disease-like pathologies. Biol Psychiatry 2010;67:522–30. 19932469 10.1016/j.biopsych.2009.09.031

[35] Wang HY, Stucky A, Liu J, Shen C, Trocme-Thibierge C, Morain P. Dissociating beta-amyloid from alpha 7 nicotinic acetylcholine receptor by a novel therapeutic agent, S 24795, normalizes alpha 7 nicotinic acetylcholine and NMDA receptor function in Alzheimer’s disease brain. J Neurosci 2009;35:10961–73. 10.1523/JNEUROSCI.6088-08.2009 PMC6665534 19726654

[36] Wang H, Lee D, D’Andrea M, Peterson P, Shank R, Reitz A. beta-Amyloid(1–42) binds to alpha7 nicotinic acetylcholine receptor with high affinity. Implications for Alzheimer’s disease pathology. J Biol Chem 2000;275:5626–32. 10681545 10.1074/jbc.275.8.5626

[37] Lauren J, Gimbel D, Nygaard H, Gilbert J, Strittmatter S. Cellular prion protein mediates impairment of synaptic plasticity by amyloid-beta oligomers. Nature 2009;457:1128–32. 19242475 10.1038/nature07761 PMC2748841

[38] Um J, Kaufman A, Kostylev M, Heiss J, Stagi M, Takahashi H, Kerrisk M, Vortmeyer A, Wisniewski T, Koleske A, Gunther E, Nygaard H, Strittmatter S. Metabotropic glutamate receptor 5 is a coreceptor for Alzheimer Aβ oligomer bound to cellular prion protein. Neuron 2013;79:887–902. 24012003 10.1016/j.neuron.2013.06.036 PMC3768018

[39] Dinamarca M, Weinstein D, Monasterio O, Inestrosa N. The synaptic protein neuroligin-1 interacts with the amyloid β-peptide. Is there a role in Alzheimer’s disease? Biochemistry 2011;50:8127–37. 21838267 10.1021/bi201246t

[40] Zheng-Fischhöfer Q, Biernat J, Mandelkow E, Illenberger S, Godemann R, Mandelkow E. Sequential phosphorylation of Tau by glycogen synthase kinase-3beta and protein kinase A at Thr212 and Ser214 generates the Alzheimer-specific epitope of antibody AT100 and requires a paired-helical-filament-like conformation. Eur J Biochem 1998;252:542–52. 9546672 10.1046/j.1432-1327.1998.2520542.x

[41] Wang J, Xia Y, Grundke-Iqbal I, Iqbal K. Abnormal hyperphosphorylation of tau: sites, regulation, and molecular mechanism of neurofibrillary degeneration. J Alzheimers Dis 2013;33:S123–39. 22710920 10.3233/JAD-2012-129031

[42] Oueslati A Implication of alpha-synuclein phosphorylation at S129 in synucleinopathies: what have we learned in the last decade? J Parkinsons Dis 2016;6:39–51. 27003784 10.3233/JPD-160779 PMC4927808

[43] Stossel T, Condeelis J, Cooley L, Hartwig J, Noegel A, Schleicher M, Shapiro S. Filamins as integrators of cell mechanics and signalling. Nature 2001;2:138–45. 10.1038/35052082 11252955

[44] Nakamura F, Stossel T, Hartwig J. The filamins: organizers of cell structure and function. Cell Adh Migr 2011;5:160–9. 21169733 10.4161/cam.5.2.14401 PMC3084982

[45] Vadlamudi R, Li F, Adam L, Nguyen D, Ohta Y, Stossel T, Kumar R. Filamin is essential in actin cytoskeletal assembly mediated by p21-activated kinase 1. Nat Cell Biol 2002;4:681–90. 12198493 10.1038/ncb838

[46] Yue J, Huhn S, Shen Z. Complex roles of filamin-A mediated cytoskeleton network in cancer progression. Cell Biosci 2013;3:7. 23388158 10.1186/2045-3701-3-7 PMC3573937

[47] Penzes P, Vanleeuwen J. Impaired regulation of synaptic actin cytoskeleton in Alzheimer’s disease. Brain Res Rev 2011;67:184–92. 21276817 10.1016/j.brainresrev.2011.01.003 PMC3109125

[48] Stucky A, Bakshi K, Friedman E, Wang H. Prenatal cocaine exposure upregulates BDNF-TrkB signaling. PLoS One 2016;11:e0160585. 27494324 10.1371/journal.pone.0160585 PMC4975466

[49] Ui N Conformational studies on proteins by isoelectric focusing. Ann N Y Acad Sci 1973;209:198–209. 4515034 10.1111/j.1749-6632.1973.tb47529.x

[50] Pace C, Grimsley G, Scholtz J. Protein ionizable groups: pK values and their contributions to protein stability and solubility. J Biol Chem 2009;284:13285–9. 19164280 10.1074/jbc.R800080200 PMC2679426

[51] Lilja A, Porras O, Storelli E, Nordberg A, Marutle A. Functional interactions of fibrillar and oligomeric amyloid-β with alpha7 nicotinic receptors in Alzheimer’s disease. J Alzheimers Dis 2011;23:335–47. 21116052 10.3233/JAD-2010-101242

[52] Zhang L, Xie J, Yang J, Cao Y. Tyrosine phosphatase STEP61 negatively regulates amyloid β-mediated ERK/CREB signaling pathways via α7 nicotinic acetylcholine receptors. J Neurosci Res 2013;91:1581–90. 24123152 10.1002/jnr.23263

[53] Snyder E, Nong Y, Almeida C, Paul S, Moran T, Choi E, Nairn A, Salter M, Lombroso P, Gouras G, Greengard P. Regulation of NMDA receptor trafficking by amyloid-beta. Nat Neurosci 2005;8:1051–8. 16025111 10.1038/nn1503

[54] Wang H, Stucky A, Liu J, Shen C, Trocme-Thibierge C, Morain P. Dissociating beta-amyloid from alpha 7 nicotinic acetylcholine receptor by a novel therapeutic agent, S 24795, normalizes alpha 7 nicotinic acetylcholine and NMDA receptor function in Alzheimer’s disease brain. J Neurosci 2009;35:10961–73. 10.1523/JNEUROSCI.6088-08.2009 PMC6665534 19726654

[55] Bloom G Amyloid-β and tau: the trigger and bullet in Alzheimer disease pathogenesis. JAMA Neurol 2014;71:505–8. 24493463 10.1001/jamaneurol.2013.5847 PMC12908160

[56] Lloret A, Fuchsberger T, Giraldo E, Viña J. Molecular mechanisms linking amyloid β toxicity and Tau hyperphosphorylation in Alzheimer׳s disease. Free Radic Biol Med 2015;83:186–91. 25746773 10.1016/j.freeradbiomed.2015.02.028

[57] Talbot K, Wang H, Kazi H, Han L, Bakshi K, Stucky A, Fuino R, Kawaguchi K, Samoyedny A, Wilson R, Arvanitakis Z, Schneider J, Wolf B, Bennett D, Trojanowski J, Arnold S. Demonstrated brain insulin resistance in Alzheimer’s disease patients is associated with IGF-1 resistance, IRS-1 dysregulation, and cognitive decline. J Clin Invest 2012;122:1316–38. 22476197 10.1172/JCI59903 PMC3314463

[58] Bateman RJ, Xiong C, Benzinger T, Fagan A, Goate A, Fox N, Marcus D, Cairns NJ XX, Blazey TM, Holtzman DM, Santacruz A, Buckles V, Oliver A, Moulder K, Aisen PS, Ghetti B, Klunk WE, McDade E, Martins RN, Masters CL, Mayeux R, Ringman JM, Rossor MN, Schofield PR, Sperling RA, Salloway S, Morris JC; Dominantly Inherited Alzheimer Network. Clinical and biomarker changes in dominantly inherited Alzheimer’s disease. N Engl J Med 2012;367:795–804. 22784036 10.1056/NEJMoa1202753 PMC3474597

[59] Trojanowski JQ, Vandeerstichele H, Korecka M, Clark CM, Aisen PS, Petersen RC, Blennow K, Soares H, Simon A, Lewczuk P, Dean R, Siemers E, Potter WZ, Weiner MW, Jack CR Jr, Jagust W, Toga AW, Lee VM, Shaw LM; Alzheimer’s Disease Neuroimaging Initiative. Update on the biomarker core of the Alzheimer’s disease neuroimaging initiative subjects. Alzheimers Dement 2010;6:230–8. 20451871 10.1016/j.jalz.2010.03.008 PMC2867838

[60] Hefter D, Kaiser M, Weyer S, Papageorgiou I, Both M, Kann O, Müller U, Draguhn A. Amyloid precursor protein protects neuronal network function after hypoxia via control of voltage-gated calcium channels. J Neurosci 2016;36:8356–71. 27511009 10.1523/JNEUROSCI.4130-15.2016 PMC6601858

